# Delirium identification among older adults hospitalized while incarcerated

**DOI:** 10.56392/001c.85001

**Published:** 2023-10-25

**Authors:** Farah Acher Kaiksow, Mustafa Quadir, Andrea Gilmore-Bykovskyi, Kaelin Rapport, Yonghe Yan, Noelle K LoConte, John Eason, Blair P Golden, Marguerite Burns

**Affiliations:** 1 Medicine, University of Wisconsin–Madison; 2 Medicine, University of Wisconsin - Madison; 3 Emergency Medicine, University of Wisconsin–Madison; 4 Anthropology, Northwestern University; 5 Sociology, Brown University; 6 Population Health Sciences, University of Wisconsin–Madison

**Keywords:** delirium, incarceration, hospital medicine

## Abstract

**Background:**

The rapidly growing population of older incarcerated patients is at increased risk of hospital-associated delirium as they have a high prevalence of comorbidities and face the unique process of accelerated aging.

**Objective:**

Our goal is to provide the first data available on identification of delirium via ICD-10 codes in this marginalized group; appropriate use of these codes communicates information across health systems and between clinicians.

**Methods:**

We examined 5,134 admissions of incarcerated patients over a 10-year period.

**Results:**

Delirium was coded in 0.4%, significantly less than in the non-incarcerated population. Those diagnosed with delirium were six times more likely to have previously been identified as cognitively impaired via ICD-10 codes.

**Conclusion:**

Incarcerated patients experience incarceration-specific care processes that increase their risk of delirium, suggesting that the rate we found is a severe underestimation. This data supports future studies aimed at assessing the true rate of and risk factors for delirium in this underserved population.

## BACKGROUND

Delirium is a common and severe condition that impacts approximately 23% of hospitalized patients, especially older adults aged 65 years and over.^1^ Unfortunately, it is often underdiagnosed by healthcare providers within the hospital setting,^2^ and even when recognized, it is frequently not documented using International Classification of Diseases (ICD) diagnosis codes.^3,4^ Proper use of ICD codes is an essential component of quality care and communication since they become a part of the medical record and aspire to uniformity across medical specialties and health care systems.^4^

Patients who are hospitalized while incarcerated in the United States may be at especially high-risk for hospital-associated delirium. This population is aging rapidly; between 1990 and 2013 the number of incarcerated individuals aged 55 and older increased by 750%.^5^ As of 2019, over 175,000 people incarcerated in US state prisons were 55 years or older.^6^ People who are incarcerated face higher baseline medical comorbidities^7^ and also experience accelerated aging, the process by which exposure to incarceration speeds up biological aging, including diseases of cognition. A 2018 study found that mild cognitive impairment (MCI) occurred far more often in incarcerated individuals, at younger ages than in non-incarcerated individuals, and persisted even after adjusting for education.^8^ The study found 78% of incarcerated people with an average age of 59 years had MCI, compared to a nationwide estimate of 10–20% of non-incarcerated people aged 65 and over.^8,9^

## OBJECTIVE

There is a significant dearth of information on delirium in adults who are hospitalized while incarcerated. This paper analyzes 10 years of data on delirium identification rates via ICD-10 codes and places the results in the context of what is known regarding the utility of ICD coding and the likely true rates of delirium among this high-risk population. This work represents a crucial first step towards fulfilling the pressing need for improving care for this underserved population, a need that has recently been brought to the forefront by both researchers and policymakers.^7,10,11^

## METHODS

We used the electronic health record (EHR) at our academic tertiary care center to identify incarcerated patients aged 18 and over who were admitted between January 1, 2010, and December 31, 2019. We included patients who were admitted for any reason, including medical, surgical, and psychiatric. Our medical center is the largest community provider of hospital care for the state’s Department of Corrections (DOC) and has over 500 admissions of incarcerated patients each year. Our hospital has a locked forensic unit where patients from DOC facilities are frequently housed during their inpatient stays; however, DOC patients and other incarcerated patients, such as those coming from local jails, can be admitted to any room in the hospital. We considered patients as hospitalized while incarcerated if a) their admission source was listed as “Court/Law Enforcement,” b) their address was a correctional facility, and/or c) their payor was the Department of Corrections. We collected data on age, sex, length of stay, location of stay (locked or non-locked unit), comorbidities, and delirium diagnoses; we considered a delirium diagnosis as present based on the use of an ICD-10 code for that admission. We calculated descriptive statistics at the stay level. We used two age cut-offs – 50 years and 65 years – to align with current practices in the field highlighting the accelerated aging associated with incarceration.^7^

## RESULTS

Between 2010 and 2019, 3,177 people were hospitalized while incarcerated for a total of 5,134 admissions (Table). The overwhelming majority were male (86%), and the average age was 46.2 years old. Delirium was diagnosed in 21 (0.4%) of all admissions). Of those 21 patients, nine were younger than 65 years. Patients diagnosed with delirium were more than 15 years older on average than those not diagnosed with delirium (62.6 vs. 46.1 years), had higher Charlson Comorbidity Index scores (5 vs. 3.7), and had much longer lengths of stay (13.4 vs. 4.8 days). Patients diagnosed with delirium were also slightly more likely to be male (90.5% vs 85.7%) and were less likely to have been hospitalized in a locked unit (24% vs. 49%). Of the 21 admissions during which delirium was diagnosed, 14 (67%) had a previous diagnosis of a cognitive disorder (delirium, altered mental status, encephalopathy, dementia, or cognitive impairment), compared to 10% of the admissions that did not include a delirium diagnosis. Further in-depth analysis was limited by the small number of delirium diagnoses among the population.

## CONCLUSION

Patients who are hospitalized while incarcerated have very low rates of ICD-10 delirium diagnosis, despite evidence suggesting that this population is likely at high-risk for hospital-associated delirium. We hypothesize that our results represent both the inadequate use of ICD codes and the impact of incarceration-specific factors and practices that are unique to the care experienced by this patient population.

Estimates of in-hospital delirium prevalence among non-incarcerated patients is around 23%,^1^ and chart review studies suggest that appropriate delirium ICD codes are used in about 46% of these cases.^12^ If we assume that the true underlying rate of delirium development and coding is similar among incarcerated patients age 65 and over, we observed only 20% of expected ICD codes. Furthermore, we expand the group at-risk for delirium to include patients 50 and older, fewer than 9% of expected patients received delirium ICD codes. As mentioned above, ICD codes are important for communicating patient information between providers and hospitals and can be used as part of the diagnostic decision-making process. This importance is supported by our findings that those patients who had previously been diagnosed with a cognitive disorder were more than six times as likely to be diagnosed with delirium during their hospitalizations, suggesting that physicians may have been more aware of the risk of delirium in these already cognitively impaired patients.

When adults are hospitalized while incarcerated, they experience incarceration-specific factors that lead to incarceration-specific care practices.^13^ These practices may diminish the likelihood of clinicians identifying delirium among incarcerated patients. Many patients who are hospitalized while incarcerated are cared for in locked units, which creates a barrier to entry and likely reduces the amount of time providers spend with them. Our data seems to support this theory, as patients diagnosed with delirium were more frequently admitted to non-locked units, which are more accessible for clinicians. Additionally, bias towards incarcerated individuals may result in reduced attention from clinicians, further decreasing the opportunities for delirium recognition. Patients hospitalized while incarcerated are less likely to have family members involved in their care, which can be vital in recognizing delirium as it represents a change from their baseline behavior. Law enforcement officials accompanying incarcerated patients typically do not know the patients well enough to provide this information, and communication with healthcare providers at correctional facilities - either through medical records or directly - is minimal.

Our study has limitations. Primarily, the small number of hospitalized incarcerated patients who were diagnosed with delirium makes comparisons difficult, although this finding itself is relevant to patient care. This was a single-site study, though the hospital is the major tertiary care center for incarcerated patients. We were unable to distinguish between different types of incarceration (long-term/prison, short-term/jail, other/temporary law enforcement custody), nor did we have information on the patients’ duration of incarceration. Unfortunately, this is a common problem in the field of incarceration and health, where many studies group together prison and jail residents for analysis.^11^ The major difference that has thus far been identified between individuals incarcerated in jails versus prisons is that those in jail are more likely to have mental health and substance use disorders.^14^

This analysis represents the first attempt at characterizing how well delirium is identified among the high-risk and growing number of older incarcerated patients. Despite limitations, the results suggest there are opportunities to improve the hospital care for older incarcerated adults. There are many reasons, including accelerated aging and increased risk factor prevalence, to suspect that age-related cognitive disorders are frequent though under-detected in incarcerated populations.^15^ The very rare use of ICD codes that we found is likely the result of both poor coding practices and, more importantly, incarceration-specific care practices that limit clinician ability to identify delirium. Efforts to better characterize the presence of age-related cognitive disorders using electronic health record-based methods are underway and should be applied to this population; increased identification and documentation of delirium is a necessary first step towards improving care.^4^ Finally, although providing acute care to patients who are incarcerated creates extra complexity for hospital medicine providers, principles of health equity require changes to our current system. As the incarcerated population continues to age, hospitals and health care organizations must work to improve the care of patients who are hospitalized while incarcerated. This data will support future studies aimed at assessing the true rate of and risk factors for delirium in this underserved population.

## Figures and Tables

**Table:** 

	All (n=5134)	Age		Delirium diagnosis status	
		<64 years (n=4571)	≥65 years (n=563)	Diagnosed with delirium (n=21)	Not diagnosed with delirium (n=5113)
**Male**	86%	85%	94%	91%	86%
**Average age, years**	46.2	43.2	70.5	62.6	46.1
**Length of stay, days**	4.9	4.8	5.6	13.4	4.8
**Admitted to locked unit**	49%	50%	46%	24%	49%
**Charlson Comorbidity Index**	3.7	3.5	4.7	5.0	3.7
**ICD-10 code for delirium present (during current hospital stay)**	0.4% (n=21)	0.2% (n=9)	2% (n=12)	(N/A)	
**Previous ICD-10 coding for delirium, AMS, encephalopathy, dementia, or cognitive impairment**	10% (n=531)	9% (n=431)	18% (n=100)	67% (n=14)	10% (n=517)
